# Associations Between Meal Program Participation and Protein Intake in People Over 65 (NHANES 2013-2018)

**DOI:** 10.1093/geroni/igab046.2338

**Published:** 2021-12-17

**Authors:** Sarah Collins, Robert Blanco, Anika Hines

**Affiliations:** 1 Virginia Commonwealth University, Richmond, Virginia, United States; 2 VCU, VCU, Virginia, United States; 3 VCU, VCU/Richmond, Virginia, United States

## Abstract

Protein plays a critical role in healthy aging. Little research exists regarding the association between meal program participation and protein consumption among individuals 65 and older. The objective of this research is to provide health professionals with a better understanding of how meal program participation through delivery services or congregate sites may relate to nutritional status. We analyzed cross-sectional data on 2845 individuals ≥65 years old who participated in the National Health and Nutrition Examination Survey (NHANES) during 2013-2018. Using linear regression models, we explored relationships between meal participation and covariates ( sex, race, marital status, income, and age) on protein intake. Protein intake did not differ significantly between individuals who participated in meal programs and those who did not. However, among individuals who answered whether or not they participated in meal programs, race was significantly associated with decreased protein intake. Non-Hispanic Blacks experienced a two-day average 8.82 grams lower [SE:1.48; p<.0001] that their white counterparts. Similarly, Hispanic/Latinos’ two-day protein average was 4.29 grams lower [SE:2.05; p=0.0426]. The association between earning an income of <$20,000 per year and protein intake was also statistically significant [β: -8.44. SE:2.4, p=0.0014]. Understanding protein intake among older adults who utilize meal programs is a gap in current literature. Results from this research may inform questions that health professionals should include in their assessments of older adults and provide guidance for nutrition policies and meal programs for people over 65.

